# Full experimental determination of tunneling time with attosecond-scale streaking method

**DOI:** 10.1038/s41377-022-00911-8

**Published:** 2022-07-07

**Authors:** Miao Yu, Kun Liu, Min Li, Jiaqing Yan, Chuanpeng Cao, Jia Tan, Jintai Liang, Keyu Guo, Wei Cao, Pengfei Lan, Qingbin Zhang, Yueming Zhou, Peixiang Lu

**Affiliations:** 1 Wuhan National Laboratory for Optoelectronics and School of Physics, Huazhong University of Science and Technology, 430074 Wuhan, China; 2 Jiangsu Key Laboratory of Micro and Nano Heat Fluid Flow Technology and Energy Application, School of Physical Science and Technology, Suzhou University of Science and Technology, 215009 Suzhou, China; 3 Optics Valley Laboratory, 430074 Hubei, China

**Keywords:** High-harmonic generation, Nonlinear optics

## Abstract

Tunneling is one of the most fundamental and ubiquitous processes in the quantum world. The question of how long a particle takes to tunnel through a potential barrier has sparked a long-standing debate since the early days of quantum mechanics. Here, we propose and demonstrate a novel scheme to accurately determine the tunneling time of an electron. In this scheme, a weak laser field is used to streak the tunneling current produced by a strong elliptically polarized laser field in an attoclock configuration, allowing us to retrieve the tunneling ionization time relative to the field maximum with a precision of a few attoseconds. This overcomes the difficulties in previous attoclock measurements wherein the Coulomb effect on the photoelectron momentum distribution has to be removed with theoretical models and it requires accurate information of the driving laser fields. We demonstrate that the tunneling time of an electron from an atom is close to zero within our experimental accuracy. Our study represents a straightforward approach toward attosecond time-resolved imaging of electron motion in atoms and molecules.

## Abstract (web summary)

A novel attosecond-scale streaking method was proposed and demonstrated to determine the tunneling time of an electron with a precision of a few attoseconds.

## Graphical abstract

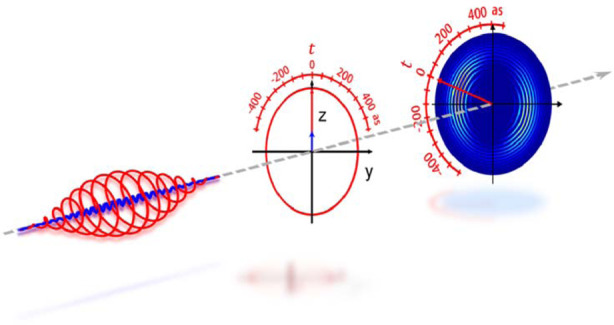

## Introduction

Timing photoionization is essential for our understanding of how light and matter interact on the most fundamental level. The advent of attosecond metrologies allows us to access the timing information on the natural time scale of electrons in atoms and molecules^1–3^. Such timing information provides the basis for our understanding of various strong-field phenomena, such as high-harmonic generation^4,5^, strong-field photoelectron holography^6,7^, and nonsequential double ionization^8,9^, which are often explained in terms of electron trajectories released at a specific ionization time.

The attoclock, or attosecond angular streaking, is a powerful tool that can access such short time scale, in which a nearly circularly polarized laser field is used to map the tunneling ionization time of an electron to its emission angle in the laser polarization plane^10^. In most of previous attoclock experiments, an offset angle between the most probable emission direction, where the ionization probability is maximum, and the minor axis of the elliptically polarized laser field was measured. It has been attempted to relate this offset angle to the time the electron spends under the tunneling barrier (tunneling time)^11–18^. The main challenge of this method comes from how to extract the tunneling time from the measured offset angle. To this end, one should firstly calculate a theoretical offset angle with assuming zero tunneling time for the attoclock, which has two significant problems. First, the offset angle is strongly affected by the ionic Coulomb potential on the electron. Thus the interpretation of attoclock experiments essentially depends on the theoretical modeling of the Coulomb effect. Second, to calculate the offset angle, one should have an accurate knowledge about the laser parameters in the experiment, e.g., the laser intensity, the laser ellipticity, and the direction of the major and minor axes of the laser ellipse. Although several proposals have been put forward to accurately calibrate those laser parameters in experiments^19–23^, the experimental conclusion is still determined by the accuracy of the theoretical calculation. So far, the question of whether the tunneling time is finite or not is still under debate and in controversy^15,18–20,24^.

Herein, we propose and demonstrate a scheme to experimentally determine the tunneling ionization time in an attoclock without any theoretical calculation. By streaking the tunneling current of an attoclock with a weak laser pulse, we resolve the tunneling ionization time relative to the field maximum in the photoelectron momentum distribution (PMD) with a precision of a few attoseconds. By directly comparing the photoelectron angular distribution (PAD) with the retrieved angular-dependent tunneling ionization time, we prove that the time interval between the instant of the momentum distribution peak and the instant of maximum field, which is often interpreted as tunneling time, is close to zero. Our method is self-referencing and independent on the theoretical modeling of the Coulomb effect on the photoelectron.

## Results

**Fig. 1:**
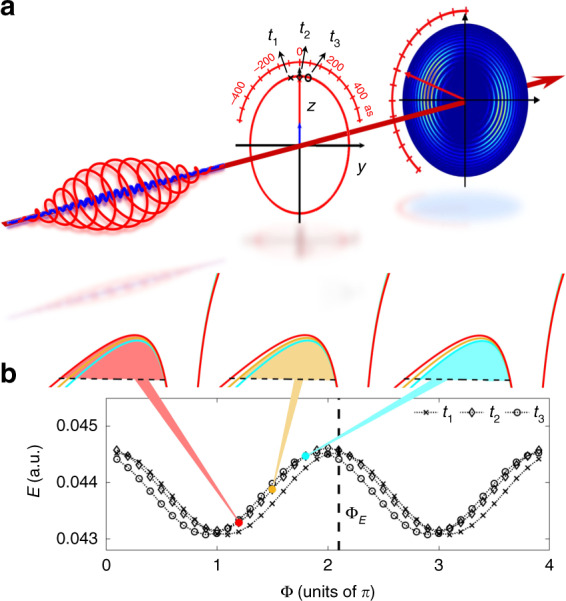
Attosecond-scale streaking scheme for measuring the tunneling ionization time. **a** An elliptically polarized 800-nm laser pulse maps the tunneling ionization time to the emission angle of the photoelectron in attoclock configurations. A perturbative SH field polarized along the major axis of the fundamental laser ellipse (*z* axis) is added to precisely determine the tunneling ionization time of the photoelectron in the momentum distribution relative to the instant of the laser-field maximum (*t*_0_ = 0). **b** The electric field strength |**E**| for three instants, as indicated in (**a**), slightly oscillates with the relative phase Φ, revealing different phase delays of Φ_**E**_, as indicated by the vertical dashed line. The variation of the electric field strength leads to a slight change of the tunneling barrier width. Due to the pronounced nonlinearity of tunneling ionization, the tunneling current will be strongly modulated by the relative phase

Figure 1 shows our method to determine the tunneling ionization time for an attoclock. In this scheme, an atom is tunnel ionized by a strong elliptically polarized 800 nm laser pulse. The instantaneous laser electric field acts as a pointer of a clock at an ionization instant *t*_0_, as shown in Fig. 1a. We add a perturbative linearly polarized second harmonic (SH) field to modulate the tunneling current produced by the strong elliptically polarized laser pulse. The two-color laser field can be written as [atomic units (a.u.) are used unless otherwise specified],

1 $$\begin{array}{*{20}{l}} {\mathbf{{{E}}}\left( t \right)} = \left[ {E_{800}\cos \left( {\omega t} \right) + E_{400}\cos \left( {2\omega t + {{\Phi }}} \right)} \right]{{{\boldsymbol{e}}}}_z \\\qquad \quad + {\it{\epsilon }}E_{800}\sin \left( {\omega t} \right){{{\boldsymbol{e}}}}_y \end{array}$$

Here *ω* is the frequency of the 800 nm fundamental field, *ϵ* is the ellipticity of the fundamental pulse, and Φ is the relative phase between the two-color components. ***e***_z_ and ***e***_*y*_ are the unit vectors along the major and minor axes of the fundamental laser ellipse, respectively. The electric field strength of the two-color field at the instant of *t*_0_ is

2 $$\begin{array}{*{20}{l}} {\left| {{{{\mathbf{E}}}}\left( {{{\Phi }};t_0} \right)} \right| = \sqrt {E_z^2 + E_y^2}}\\\qquad\qquad\ = \left| {{{{\mathbf{E}}}}_0\left( {t_0} \right)} \right| + \xi E_1\cos \left( {{{\Phi }} + 2\omega t_0} \right) + O\left( {\xi ^2} \right) \end{array}$$

where $\left| {{{{\mathbf{E}}}}_0\left( {t_0} \right)} \right|$ is the electric field strength of the 800 nm field, $E_1 = E_{800}\cos \left( {\omega t_0} \right)/\sqrt {{\it{\epsilon }}^2{{{\mathrm{sin}}}}^2\left( {\omega t_0} \right) + {{{\mathrm{cos}}}}^2(\omega t_0)}$, and $\xi = E_{400}/E_{800}$ is a small parameter characterizing the ratio of the two fields. Due to the perturbative nature of the SH field, we can neglect the small higher-order terms in Eq. (2). Thus, the electric field strength at *t*_0_ oscillates with the relative phase of the two-color fields, which maximizes at the relative phase of

3 $$\begin{array}{*{20}{c}} {{{\Phi }}_{{{\mathrm{E}}}}\left( {t_0} \right) = - 2\omega t_0} \end{array}$$

Because the width of the tunneling barrier approximately scales as $I_p/\left| {{{{\mathbf{E}}}}\left( {{{\Phi }};t_0} \right)} \right|$ with *I*_*p*_ being the ionization potential, the tunneling barrier width at time *t*_0_ also changes with the relative phase. The exponential decay of the wave function in the barrier means that small variations of the electric field strength will lead to a large change of the ionization probability. As a result, the electron yield for a continuum state **p** will be strongly modulated by the relative phase Φ. If the oscillation of the electron yield for a continuum state **p** is in phase with the oscillation of the electric field strength at the instant of *t*_0_, this means that the electron of the continuum state **p** is released at the instant of *t*_0_. Therefore, the tunneling ionization time in the PMD can be precisely determined by scanning the relative phase of the two-color laser field.

**Fig. 2:**
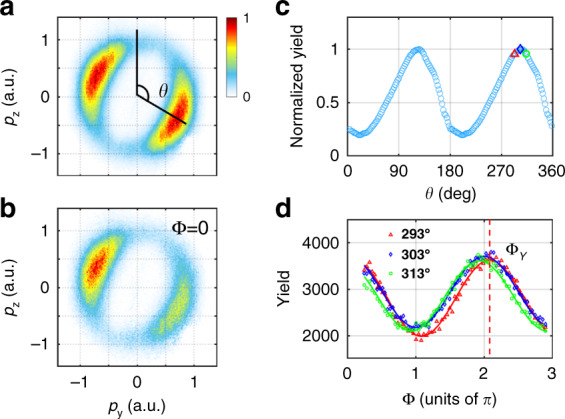
Measured PMDs and electron yields. The measured PMDs in the polarization plane (**a**) for an average of all relative phases and (**b**) for the relative phase of zero. The electron emission angle *θ* is defined between the electron emission direction relative to the major axis of the elliptically polarized laser field. **c** The radially integrated PAD of (**a**). **d** The electron yield as a function of the relative phase for three emission angles (293°, 303°, and 313°) in angular bins of 1°. The solid lines in (**d**) are the fit of the experimental data. ΔΦ_*Y*_ for the emission angle of 293° is indicated by the dashed vertical line

The measured PMDs are shown in Fig. 2a, b for an average of all relative phases and for the relative phase of zero, respectively. The major and minor axes of the fundamental laser ellipse are nearly along the *p*_*z*_ and *p*_*y*_ directions, respectively. Because of the perturbative nature of the SH field, the PMD in the two-color laser field with an average of all relative phases is nearly the same as that in a one-color fundamental field (see Supplementary Material). We see that the PMD is symmetric with respect to the origin in Fig. 2a, while it becomes asymmetric for the relative phase of zero in Fig. 2b. The radially integrated PAD of Fig. 2a is shown in Fig. 2c, where the most probable emission angle (the peak of the PAD) appears at 123° and 303°. The most probable emission angles deviate significantly from the prediction of the strong-field approximation (90° and 270°)^25–27^. The difference between the most probable emission directions relative to the minor axis of the fundamental laser ellipse is often referred to as the offset angle. Here, we determine the ionization time using the oscillation of the electron yield with the relative phase for each emission angle instead of the offset angle. In Fig. 2d, the yields for three emission angles are shown as a function of the relative phase. We can clearly see that the yield in each emission angle oscillates obviously with the relative phase with a large amplitude, though the streaking field is very weak. This comes from the fact that the tunneling current depends exponentially on the barrier width and therefore on the electric field strength of the laser. By fitting the oscillations with the function of $Y = Y_0 + Y_1{\rm cos}\left( {{{\Phi }} - {{\Phi }}_Y\left( {{{\mathbf{p}}}} \right)} \right)$ ^28,29^, where *Y*_0_ and *Y*_1_ are two variables independent on the relative phase, we can obtain the phase delays Φ_*Y*_ (**p**) for different emission angles.

According to the scheme in Fig. 1, the tunneling ionization time is determined if the oscillation of the electron yield with respect to Φ is in phase with the oscillation of the electric field strength at the instant of *t*_0_, i.e., ${{\Phi }}_Y\left( {{{\mathbf{p}}}} \right) = {{\Phi }}_E\left( {t_0} \right)$. As a result, the tunneling ionization time for the continuum state **p** can be obtained by,

4 $$\begin{array}{*{20}{c}} {t_0({\mathbf{p}}) = - {{\Phi }}_Y\left( {{{\mathbf{p}}}} \right)/\left( {2\omega } \right)} \end{array}$$

**Fig. 3:**
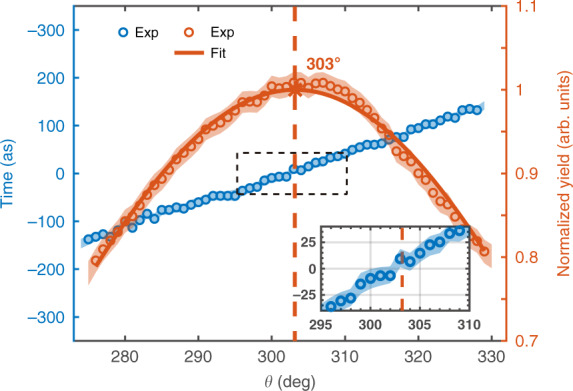
Measured tunneling ionization time with respect to the electron emission angle. For comparison, the radially integrated PAD is shown by the red circles, which is fitted with a Gaussian function (red solid curve) determining the peak of the PAD (vertical dashed line). The inset shows the enlarged view of the region inside the dashed frame. Note that zero time corresponds to the field maximum of the elliptically polarized laser pulse. The experimental errors (shaded area) for the time show the 95% confidence interval for the fitting process, and those for the photoelectron yield show the standard deviation of the statistical errors

The mapping relation between the phase delay and the ionization time in Eq. (4) is the basis of extracting the tunneling ionization time from the measurement. Thus, we can determine the tunneling ionization time for different emission angles, as shown by the blue dots in Fig. 3. One can see that the tunneling ionization time extracted from the measurement depends linearly on the emission angle within (270°, 330°), which agrees with the prediction of the time-to-angle mapping relation according to the attoclock principle^11,18^. To obtain the information about the tunneling time, we further show the PAD in Fig. 3 for comparison. By fitting the PAD with a Gaussian function, we see that the most probable emission angle appears at 303°, which almost coincides with the zero of time (the instant of maximum field), as shown by the red dashed line. This means that the zero of time in the attoclock corresponds to the most probable emission angle in the PMD, in agreement with theoretical predictions^17,30,31^. It is noteworthy that the time interval between the instant of the most probable emission angle and the instant of the maximum field is often interpreted as the tunneling time. Here our experiment unambiguously demonstrates that this time interval is very close to zero, which invalidates the long tunneling time inferred from some previous studies^12,14,18,20,32^.

## Discussion

**Fig. 4:**
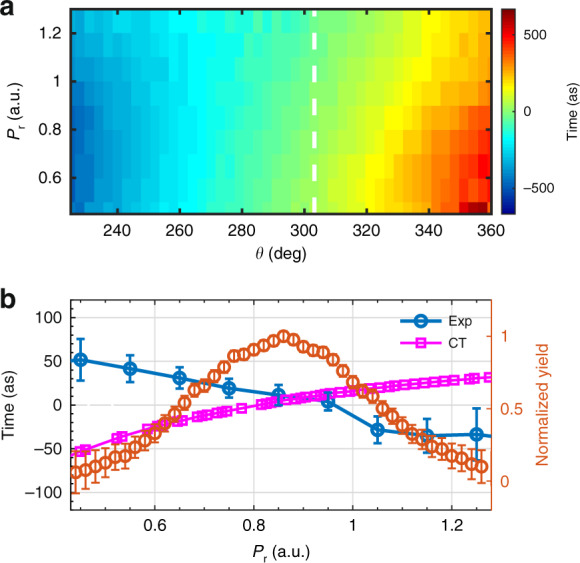
Energy- and angle-resolved tunneling ionization time. **a** The extracted tunneling ionization time with respect to the electron momentum *p*_*r*_ and the electron emission angle from the measurement. **b** The lineout taken at the emission angle of 303° from (**a**). The prediction by the classical-trajectory (CT) model is shown by the squares. For comparison, the normalized yield as a function of the electron momentum at *θ* = 303° is shown by the red circles. The error bars for the ionization time show the 95% confidence interval for the fitting process, and those for the photoelectron yield show the standard deviation of the statistical errors

Using the present scheme, we can further retrieve the ionization time of electrons with different energies^33^. Figure 4a shows the extracted tunneling ionization time with respect to the electron momentum $p_r = \sqrt {p_z^2 + p_y^2}$ and the electron emission angle from the measurement. To study the energy dependence of the tunneling ionization time, we show in Fig. 4b the lineout taken from Fig. 4a at the emission angle of 303°. We find that the extracted tunneling ionization time from the measurement at the most probable emission angle decreases with increasing electron energy.

The energy-dependent tunneling ionization time can be calculated by a simple classical-trajectory (CT) model. As shown by the squares in Fig. 4b, the tunneling ionization time predicted by the CT model increases with increasing *p*_*r*_. This can be interpreted remarkably simply. The less energetic photoelectrons (smaller *p*_*r*_) spend more time interacting with the ion, thus the Coulomb effect is more significant for those electrons. As a result, those electrons should be released earlier to maintain the same emission direction as the more energetic photoelectrons. Surprisingly, the measurement contradicts with the prediction of the CT model. The difference between the experiment and the CT simulation might come from non-stationary under-the-barrier electron dynamics, which should have a significant influence on the initial momentum distribution at the tunnel exit^34,35^. We hope that the difference between the measurement and the CT simulation would stimulate much theoretical interest on studying the under-the-barrier dynamics in strong-field tunneling ionization.

It is worth noting that, comparing to previous attoclock experiments^11,12,14,15,19,20^, our temporal scheme has at least two advantages. First, the Coulomb effect on the electron has been naturally excluded in our scheme. In previous attoclock experiments, one should firstly accurately remove the Coulomb effect from the measured offset angle via theoretical calculation. This process usually depends on a specific model, leading to some model-dependent conclusions^11,12,14,15,19,20^. In the present scheme, the tunneling ionization time is extracted from the phase delay of the oscillation of the electron yield, which is not influenced by the Coulomb effect. Second, the retrieved tunneling time in our scheme is independent of accurate knowledge of the laser parameters in the experiment. To calculate the offset angle in previous attoclock experiments, one should accurately calibrate the laser parameters in the experiment. In our scheme, the time interval between the instant of the momentum distribution peak and the instant of maximum field is obtained by directly comparing the PAD and the phase delay for each emission angle, as shown in Fig. 3. Both PAD and phase delay are direct experimental observables. Thus our result is obtained without relying on the accurate knowledge of the laser parameters in experiments, such as the laser intensity and ellipticity.

In summary, we have determined the tunneling ionization time in the PMD with a precision of a few attoseconds. This is achieved by a perturbative laser pulse to modulate the tunneling current produced by a strong elliptically polarized laser field in attoclock configurations. By comparing the PAD with the extracted angular-dependent tunneling ionization time, we demonstrate that the time required for an electron to tunnel through a potential barrier is close to zero for an atom within our experimental accuracy. This means that the offset angle in the attoclock does not come from the tunneling time delay. Instead, it is relevant to the Coulomb effect of the ionic core or the possible multi-electron interaction. Furthermore, we find that the tunneling ionization time at the most probable emission angle is shifted to an earlier ionization moment with increasing the electron energy, which contradicts with the prediction of the classical-trajectory model. This remains an interesting topic for further investigation. Our method is self-referencing and independent of theoretical modeling of the Coulomb effect. Extending our method to molecules and even solids can provide us not only the fundamental dynamics of laser–matter interaction but also the potential of retrieval of geometrical information of the targets.

## Materials and methods

### Experimental methods

Our experiment used laser pulses that are centered at 800 nm with ~40 fs duration and a repetition rate of 5 kHz. The laser pulses were propagated through a 300-µm-thick *β*-barium borate (*β*-BBO) crystal for SH generation. After the BBO, the laser pulse consisted of both fundamental and SH fields. A wire grid polarizer combined with a two-color wave plate was used to adjust the intensity ratio of the two-color laser field. The two-color laser pulse then passed through two dual-order wave plates that change the polarization of the fundamental component while keeping that of the SH component unchanged. The relative phase between the two-color components was controlled by a pair of glass wedges, one of which was mounted on a motorized delay stage. The absolute value of the relative phase was calibrated from the measured most probable emission angle as a function of the wedge position (see Supplementary Material for details). The two-color laser pulse was then focused into the supersonic atomic beam with a parabolic mirror (*f* = 75 mm) to ionize the Ar atoms. The three-dimensional momenta of the resulting photoelectrons were detected using cold target recoil ion momentum spectroscopy (COLTRIMS)^36,37^. The ellipticity and the intensity of the fundamental laser field were calibrated to be 0.88 and 1.2 × 10^12^ Wcm^−2^, respectively. The intensity ratio between the SH and fundamental fields is estimated to be ~1/6400. It should be noted that the maximal vector potential of the SH field is only *E*_400_/2ω = 0.006 a.u., thus the SH field only slightly changes the width of the tunneling barrier and has a negligible contribution to the electron trajectory.

### Classical-trajectory model

We use a simple classical-trajectory model to understand the energy-dependent tunneling ionization time of the electron. In this model, the electron is released at the tunnel exit position of $I_p/\left| {{\mathbf{E}}\left( t \right)} \right|$ with zero initial momentum longitudinal to instantaneous tunnel direction^38^. Then the electron trajectory is propagated in the combined laser and Coulomb fields by numerically solving the classical Newtonian equation. Depending on the initial momentum transverse to the tunnel direction and the tunneling ionization time, the electron will be emitted to different directions and with different *p*_*r*_. By selecting those electrons emitted along the most probable emission angle, we obtain the dependence of the tunneling ionization time on the electron energy.

## Supplementary information

Supplementary Material

## Data Availability

All data needed to evaluate the conclusions in the paper are present in the paper and/or the Supplementary Materials. Data from these experiments and codes used for data analyses are available from the corresponding authors upon reasonable request.
