# Supplementary material for: Full experimental determination of tunneling time with attosecond-scale streaking method
Source: Light Sci Appl. 2022 Jul 7;11:215. doi: 10.1038/s41377-022-00911-8 (PMC9262890; doi:10.1038/s41377-022-00911-8)
Supplement: Supplementary file 1 — Supplementary Material [file 41377_2022_911_MOESM1_ESM.pdf]

Supplementary information for

Full experimental determination of tunneling time with attosecond-scale streaking method

Miao Yu, Kun Liu, Min Li, Jiaqing Yan, Chuanpeng Cao, Jia Tan, Jingtai Liang, Keyu Guo, Wei Cao, Pengfei Lan, Qingbin Zhang, Yueming Zhou, and Peixiang Lu

1. Comparison of phase-averaged PMD in the two-color laser field and the PMD in the single-color laser field

In this work, we use a weak perturbative 400 nm field to streak the tunneling current produced by a strong elliptically polarized laser field. Because the intensity ratio between the 400 nm and 800 nm fields is weak enough ($\sim$1/6400), the photoelectron momentum distribution (PMD) in the two-color laser field with an average of all relative phases is nearly the same as that in the single-color 800 nm field. To demonstrate this point, we have calculated the PMD in the two-color laser field as well as that in the single-color 800 nm laser field by solving the time-dependent Schrödinger Equation (TDSE). The results are shown in Fig. S1, in which the PMD in the two-color field with an average of all relative phases is shown in Fig. S1(a) and the PMD in the single-color 800 nm laser field is shown in Fig. S1(b). One can see that the PMDs in Figs. S1(a) and S1(b) are nearly the same. Particularly, the radially integrated photoelectron angular distributions (PADs) of (a) and (b) are shown in (c) by the circles and blue line, respectively. We also see that the PAD in the two-color laser field with an average of all relative phases agrees quantitatively with that in the single-color 800 nm field due to the perturbative nature of the 400 nm laser field.

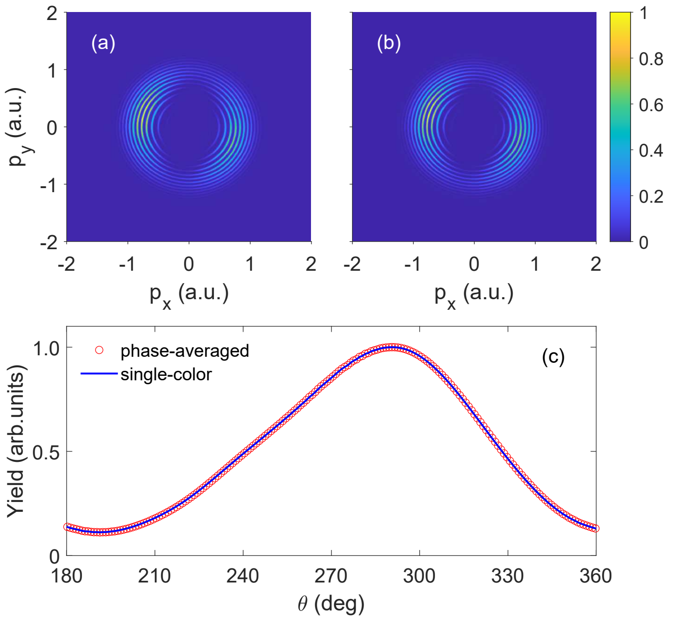

Figure S1: (a) and (b) show the PMD calculated by the TDSE for an average of all relative phases in the two-color laser field and for the single-color 800 nm laser field. The radially integrated PADs of (a) and (b) are shown in (c) by the circles and blue line, respectively. The intensity of the 800 nm laser field is the same for those two cases.

2. Calibrating the zero of the relative phase of the two-color laser field

In our experiment, the relative phase of the two-color field was controlled by a pair of glass wedges, one of which was mounted on a motorized delay stage. The relative phase between the two-color field is linear to the wedge position, i.e., $\Phi = ax + b$, we need to determine the constants a and b. In the experiment, we have continuously scanned the wedge position by a motorized delay stage and recorded its value for each event.

In Fig. S2(a), we show the radially integrated PAD with respect to the wedge position. The PAD for each relative phase has been normalized to its maximum yield. One can see that the most probable emission angle θ obviously oscillates as a function of the wedge position, as guided by the white solid line. From the period of this feature, we obtain the constant a. The phase offset b is determined when the most probable emission angle coincides with the most probable emission angle in the single-color 800 nm laser field, as indicated by the black vertical lines in Fig. S2(b). The reason is as following.

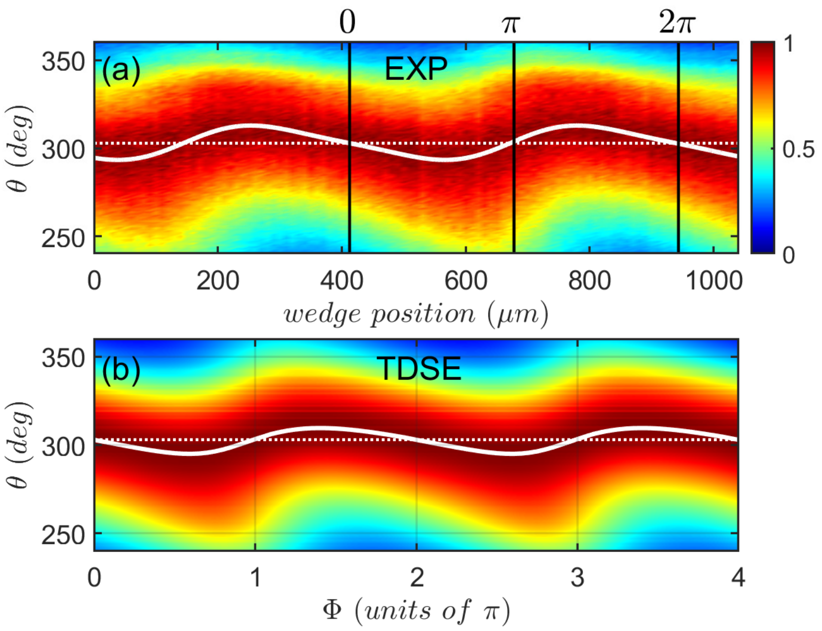

Figure S2: (a) The measured PAD as a function of the wedge position. Here the PAD for each wedge position has been normalized to its maximum yield. The white solid line is used to guide the oscillation of the most probable emission angle with the wedge position. The white dashed line shows the most probable emission angle for the single-color 800 nm laser field. The black vertical lines represent the relative phases extracted from the plot. (b) The PAD as a function of the relative phase calculated by the TDSE. The white solid line is used to guide the oscillation of the most probable emission angle with the relative phase. The white dashed line shows the most probable emission angle for the single-color 800 nm laser field.

In the attoclock experiments, the offset angle $\triangle\theta$ has two contributions,

$$\triangle\theta = \theta_t + \theta_c, \tag{S1}$$

where θ_t is due to the tunneling time delay (if there is nonzero tunneling time delay) and θ_c results from the coulomb interaction between the parent ion and the escaping electron. When a weak second harmonic field is added, the direction of combined electric field maximum oscillates around the major axis of the elliptically polarized fundamental field, as shown in Fig. S3. It introduces an additional contribution to the offset angle,

$$\triangle\theta = \theta_t + \theta_c + \theta_F(\Phi), \tag{S2}$$

where Φ is the relative phase. $\theta_F(\Phi)$ is solely due to change of the direction of combined electric field maximum by the perturbative field (this direction

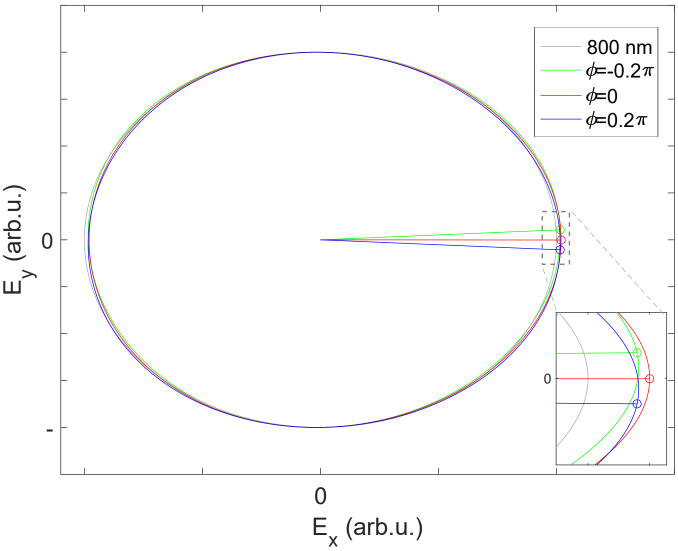

Figure S3: The electric field of the laser pulses. The gray line shows the electric field of the fundamental 800 nm pulse. The green, red and blue lines show the electric field of the 800 nm + 400 nm pulses with different relative phases. At the relative phase Φ=0, the direction of the electric field maximum coincides with that of the 800 nm field (i.e., along the x-axis).

could be considered as the "major axis" of the combined field). So, the most probable emission angle oscillates with the relative phase Φ, as shown in Fig. S2(a). Because the intensity of the second harmonic field is several orders of magnitude lower than the fundamental field, it neither changes the tunneling time delay (i.e., θ_t), nor the coulomb interaction between the parent ion and the escaping electron (i.e., θ_c, see Sec. 3 below). The oscillation of the most probable emission angle is exclusively due to $\theta_F(\Phi)$ in Eq. (S2). At the relative phase $\Phi = 0$, the direction of the electric field maximum coincides with that of the fundamental field (as shown in Fig. S3). So, the most probable emission angle should coincide with that of the fundamental 800 nm field. Therefore, in our experimental, we trace the most probable emission angle at different relative phases and compare it with that of the fundamental 800 nm field. The absolute value $\Phi = 0$ is determined when the most probable emission angle coincides with the fundamental 800 nm field, as shown in Fig. S2(a). Here, we don't rely on any theoretical calculation. The most probable emission angle in the experiment is obtained by fitting the photoelectron angular distribution with a Gaussian function. The fitting process leads to a measurement error of $\sim 0.5°$ for the most probable angle, corresponding to ~ 4 as (95% confidence interval) for the ionization time.

To further validate this calibration process, we also numerically solve the TDSE and obtain the PAD as a function of the relative phase. As

shown in Fig. S2(b), the TDSE-calculated most probable emission angle at the relative phase of $\Phi = k\pi$ (k is an integer) indeed coincides with that of the single-color 800 nm field. This means that the zero of the relative phase can be directly obtained from the experimental data without relying on theoretical calculation.

3. The effect of the 400 nm field on the motion of the escaping electron

In our experiment, the intensity of the 400 nm field is three order of magnitude lower than that of the fundamental field. Therefore, the 400 nm field only modulates the ionization rate and slightly changes the electron trajectory. The change of electron motion in the continuous state induced by the second harmonic is negligible, as shown in Fig. S4. The angular shift induced by the 400 nm field is less than 0.5 degree for all relative phases.

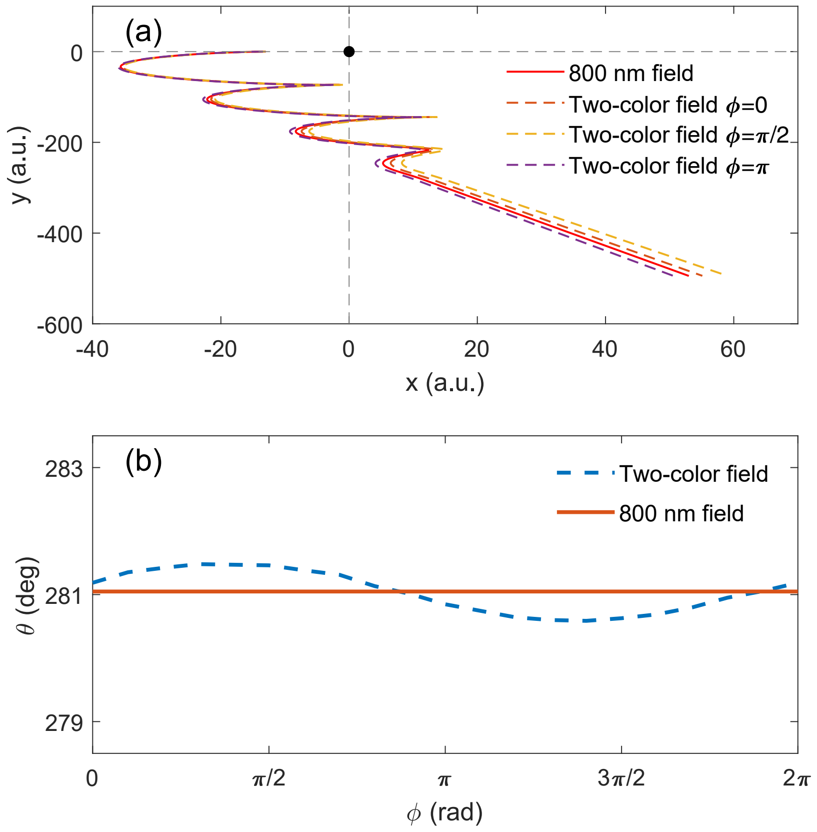

Figure S4: (a) The trajectories of the tunneled electron. (b) The angular shift induced by the second harmonic as a function of the relative phase. The red line indicates the most probable emission angle in the fundamental 800 nm field.
